# Wuxal amino (Bio stimulant) improved growth and physiological performance of tomato plants under salinity stress through adaptive mechanisms and antioxidant potential

**DOI:** 10.1016/j.sjbs.2021.04.040

**Published:** 2021-04-24

**Authors:** Mohamed M. Ali, Kaouthar Jeddi, Mohamed S. Attia, Salah M. Elsayed, Mohammad Yusuf, Mahmoud S. Osman, Mona H. Soliman, Kamel Hessini

**Affiliations:** a Horticulture Research Institute, Agricultural Research Center, Giza, Egypt; b Laboratory of Plant Biodiversity and Dynamic of Ecosystems in Arid Area, Faculty of Sciences of Sfax, B.P. 1171, Sfax 3000, Tunisia; c Botany and Microbiology Department, Faculty of Science, Al-Azhar University, Nasr City 11884, Cairo, Egypt; d Biology Department, College of Science, United Arab Emirates University, Al Ain 15551, United Arab Emirates; e Botany and Microbiology Department, Faculty of Science, Cairo University, Giza 12613, Egypt; f Department of Biology, College of Sciences, Taif University, P.O. Box 11099, Taif 21944, Saudi Arabia

**Keywords:** Wuxal amino, Secondary metabolites, Antioxidants, Oxidative stress, Tomato

## Abstract

In the present study, ameliorative capabilities of wuxal amino (bio stimulant) under salt stress has been investigated through adaptive mechanisms and antioxidant potential in tomato plants. In the experiment, two different concentrations (2 cm L^-1^ and 3 cm L^-1^) of wuxal amino through foliar application and soil irrigation were applied to the salt (150 mM) treated tomato plants and then morphological traits, photosynthetic pigments, osmolytes, secondary metabolites, oxidative stress and antioxidant enzymes activity were assessed at 60 days after planting. The results revealed that salt stress decreased the growth parameters, photosynthetic pigments, soluble sugars and soluble protein whereas, content of proline, ascorbic acid, total phenols, malondialdehyde, hydrogen peroxide and the activity of antioxidant enzymes activity increased under salt stress. Moreover, Wuxal amino application through foliar or soil to salt stressed plants improved morphological traits, photosynthetic pigments, osmolytes, total phenol and antioxidant enzymes activity. Interestingly, the deleterious impact of salinity on tomato plants were significantly reduced and it can be evident from reduced MDA and H_2_O_2_ levels. These responses varied with the mode (foliar or soil) of application of Wuxal amino under different concentrations (2 cm L^-1^ and 3 cm L^-1^). It was concluded that application of Wuxal amino (2 cm L^-1^, foliar) and (3 cm L^-1^; soil) proved best and could be commercially used as eco-friendly tool for the protection of tomato plants grown under salinity stress.

## 1 Introduction

One of the most daunting challenges for farmers is to feed the ever-increasing global population, that is currently growing at a rate of about 1.05% per year, under the increasingly globalized climatic and natural disturbances (Loudière and Gourbesville, 2020). To cope with these challenges, researchers estimated that by 2050, it might be necessary to shift the maximum output of important food crops by about 87 percent (Fróna et al., 2019, Loudière and Gourbesville, 2020). As these climatic conditions are often linked with major abiotic constraints including drought, heat, cold, and salt stress that cause significant loss to plant growth, production, yield and food quality (Ahanger et al., 2018, Ahmad et al., 2018a, Soliman et al., 2019, Kaya et al., 2020, Kononenko et al., 2020, Soliman et al., 2020a). Among the abiotic stresses, soil salinization is most detrimental to crops in terms of plant growth, development, and ultimate crop productivity and food security (Ahmad et al., 2014, Kumar et al., 2015, Sharma et al., 2016, Acosta-Motos et al., 2020, Soliman et al., 2020a). The soil salinization issues are increasing by about 20%, nationally and globally, for the agricultural land and this increase is continuous (Gupta and Huang, 2014, Gharsallah et al., 2016). As a consequence, for maintaining global food security and minimizing economic losses it is necessary to understand crop resilience against multiple stresses in order to optimize better yields and reduce agronomic performance (Gupta and Huang, 2014, Gupta et al., 2015).

Salinity stress also indirectly induces the accumulation of ROS, such as singlet oxygen, superoxide radicals, and H_2_O_2_ (Ahmad et al., 2018b, Mir et al., 2018). Increased oxidative stress limits vital processes such as transpiration (Abdallah et al., 2020), water absorption and nutrient uptake dynamics as well as chlorophyll biosynthesis (Cendrero-Mateo et al., 2015, Rizwan et al., 2015, Ma et al., 2020) which collectively cause reduction in plant growth and yield (Sehar et al., 2019, Alhaithloul et al., 2020, Senousy et al., 2020). Plants have developed adaptive responses to survive under salt stress which include development of morphological, physiological and metabolic adaptations (Shahid et al., 2020). Improvements of photosynthetic machinery and the accumulation of osmoprotectants may be useful strategies and may play a supportive role in preserving salt-induced changes (El-Beltagi et al., 2020, Osman et al., 2020). Nevertheless, antioxidant defence modulation, either enzymatic or non-enzymatic (Latif and Mohamed, 2016, Agathokleous, 2020, Senousy et al., 2020, Soliman et al., 2020b) also play a crucial role in alleviating salt-induced oxidative stress.

Over-accumulation of ions such as calcium (Ca^2+^), magnesium (Mg^2+^), sodium (Na^+^), sulphates (SO4^2−^), and chlorides (Cl^-^) with a special abundance of Na^+^, creating soil salt toxicity. The accumulation of these ions increase alkalinity and creating a problem associated with soil salinity in Egypt and therefore induce osmotic, oxidative and ion stress that lead to destructive cellular activity (Naveed et al., 2020). To date, many management activities have suggested to enhance the salt tolerance underlying mechanisms, however, environment-friendly approaches and successful use of treatments is an innovative strategy to ensure crop yields in such stressful circumstances (AbdElgawad et al., 2016, Singh et al., 2018) much needed.

Several studies evaluating the impact of organic or synthetic chemical materials as effective strategies to minimize salinity-induced damage so as to increase the crop production and ability to cope with stresses (Yakhin et al., 2017, Carillo, 2018). Bio-stimulants are kind of bioactive molecules with rich ingredients and seeking eco-friendly and sustainable ways to promote plant growth and development when applied in a small amount (Kumar et al., 2015, Xu and Geelen, 2018). Bio-stimulants increase soil nutrient absorption and improve nutrient quality and thereby contributing to growth improvement and stress tolerance in plants (Nephali et al., 2020). Wuxal Amino meets the requirements of the European Union for admission as working fonds of ecological farming. Previous research is mixed in terms of the impact of Wuxal Amino, as a biofertilizer containing NPK and 9% organically fixed nitrogen and many more effective amino acids forms (proline, alanine, glycine and threonine). In addition, WUXAL Amino contains a variety of different amino acids forms, could effectively enhance the vegetative growth of woody plants (Pfeiffer et al., 2008, Szabó et al., 2014).

Tomato (*Solanum lycopersicum* L.) is a major vegetable crop affected by various abiotic stresses throughout the globe. It is well known for its edible fruits as a rich source of antioxidants, phytochemical, antimicrobial and anti-inflammatory contents (Chaudhary et al., 2018). Tomato fruit is rich in vitamin C, vitamin A and energy (Raiola et al., 2014, Ayenan et al., 2019). Tomato is a moderately salt-tolerant crop mostly cultivated in areas with cool and dry climatic conditions (Raza et al., 2017). Salt stress has been reported to have significant impact on the growth, physiology and yield of tomatoes (Bacha et al., 2017). Keeping above reports in mind, present study was designed to dissect the effect of wuxal amino on growth performance of tomato plants through different mode (foliar and root) of application and also investigate wuxal amino mediated amelioration of salt stress in tomato plants through modulating vegetative growth and physiological traits.

## 2 Materials and methods

### 2.1 Experimental site and bio-stimulant treatment

The experiment was conducted at the experimental farm of AL-SALAM International for Development & Agriculture Investment, Egypt. Wuxal Amino as a bio-stimulant obtained by AL-SALAM International for Development & Agriculture Investment, Egypt from Aglucone Fertilizers GmbH & Co. KG (AGLUKON Spezialdünger GmbH & Co.KG), Düsseldorf, Germany. Wuxal® Amino contain a mixture of nitrogen and amino acids as follows: Total nitrogen 110 g^−1^/L, total amino acids (mainly proline, alanine, glycine and threonine) 648 g^−1^/L and pH value 7.0.

### 2.2 Experimental design

Four week old tomato seedlings (*Solanum lycopersicum* L. *var*. 023) were obtained from Agriculture Research Centre, Giza, Egypt. Uniform seedlings were transplanted into plastic pots (40 × 40 cm) containing mixture of sand and clay (1: 3), total 7 kg, in a plastic greenhouse. Pots were kept in the greenhouse maintained at 22/18 °C day/night temperature and 70–85% relative humidity. After transplant, plants were irrigated normally for five days. Thereafter, salt solution (150 mM NaCl) was administered three times (after 5 days gap). The wuxal amino was applied for three times (once in a week) before and after flowering. Wuxal was given either through foliage or soil. The details of treatments include set I-control; set II- 150 mM NaCl; set III- NaCl + wuxal amino (2 cm /L, through root irrigation); set IV- NaCl + wuxal amino (2 cm /L, though foliar); set V- NaCl + wuxal amino (3 cm /L, through root irrigation) and Set VI- NaCl + wuxal amino (3 cm /L, though foliar). Sixty days after planting (60 DAP) plants were carefully uprooted and analysed for the different parameters described below.

### 2.3 Vegetative growth parameters

Growth parameters including shoot fresh weight and root fresh weight were estimated immediately after harvesting. Dry weight of shoots and roots was determined by oven-drying samples at 70 °C for 24 h. Moreover, plant height (cm^−1^), root length (cm^−1^) and number of leaves per plant were also recorded.

### 2.4 Photosynthetic measurements

For estimation of pigments fresh 0.5 g leaf tissue was ground in acetone (80%) using pestle and mortar. After centrifuging for 5 min at 10,000 g absorbance of filtrate was measured at 470, 652 and 665 nm to estimate chlorophyll *a*, chlorophyll *b* (Vernon and Seely, 1966) and carotenoid (Lichtenthaler and Buschmann, 2001).

### 2.5 Estimation of stress biomarkers

#### 2.5.1 Lipid peroxidation

Malondialdehyde (MDA) content was measured using the thiobarbituric acid (TBA) method according to Heath and Packer, 1968, Kamyshnikov, 2002 with slightly modification. The MDA content was determined according to its molar coefficient of absorbance of 155 mmol ·L^−1^ ·cm^−1^ and expressed as nmolg^−1^ FW.

#### 2.5.2 Hydrogen peroxide (H_2_O_2_) content

Hydrogen peroxide levels were determined according to Velikova et al. (2000). Fresh leaf was homogenised in 2 mL of 0.1% trichloroacetic acid (TCA) solution. After centrifugation at 12,000 × g for 15 min, 0.5 mL of the supernatant was added to the reaction mixture containing 0.5 mL of 10 mM K phosphate buffer (pH 7.0) and 1 mL of 1 M KI. Absorbance was determined at 390 nm. The blank was prepared in the same manner except that 1 mL of 10 mM K phosphate buffer (pH 7.0) instead of the sample. The amount of H_2_O_2_ was calculated from calibrated samples using (1, 5, 10 mM H_2_O_2_) standard solutions, each standard solution was added to the reaction mixture containing 0.5 mL 10 mM K phosphate buffer (pH 7.0) and 1 mL of 1 M KI. Absorbance was determined at 390 nm.

#### 2.5.3 Estimation of total phenols

Total phenolic content was determined using the process described by Dai et al. (1993), with minor modifications. A 100 μL extract volume was added to the 1.5 mL Folin–Ciocalteu reagent solution and incubated at room temperature for 1 min. Subsequently, 1.5 mL of sodium carbonate solution was added and left at room temperature for 90 min in the dark. Absorbance was checked at 765 nm. Total phenolic content was determined by the gallic acid calibration curve and expressed as mg g^−1^ dry weight.

#### 2.5.4 Determination of the content of osmolytes (Total Soluble Protein, Proline and Soluble Sugar)

Content of soluble protein was estimated following Lowry et al. (1951) using Folin phenol reagent and absorbance was recorded at 700 nm using bovine serum albumin as standard. Method of Bates et al. (1973) was used for estimation of proline. Briefly, 0.5 g dried leaves were extracted in 3% sulphosalicylic acid. After centrifugation at 10.000g for 10 min, supernatant was mixed with ninhydrin reagent and absorbance was taken at 520 nm. For measuring soluble sugar content, anthrone method was used and absorbance was measured at 625 nm (Irigoyen et al., 1992).

#### 2.5.5 Estimation of ascorbate (Ascorbic acid content)

The ascorbic acid (AsA) was determined according to Jagota and Dani (1982). Leaf samples (0.2 g) were ground with liquid N_2_ and suspended in 2 mL of 5% TCA. The homogenate was centrifuged at 10,000*g* for 15 min at 5 °C. AsA extraction solution was mixed with 10% TCA which was vigorously shaken and then placed in an ice bath for 5 min. 0.5 mL of the extract was diluted to 2.0 mL using double distilled water, and 0.2 mL of diluted Folin-Ciocaiteu reagent was added to the previous mixture, and the absorbance of the blue colour developed was measured after 10 min at 760 nm. The AsA content was calculated using a standard curve of ascorbic acid.

#### 2.5.6 Antioxidant enzymes assay

Fresh tomato (1.0 g) leaves were extracted in 100 mM phosphate buffer (pH 7.8) containing PVP and EDTA where the homogenate was centrifuged at 15,000*g* for 10 min and the supernatant was used for assaying enzyme activity. The activity of superoxide dismutase (SOD; EC 1.15.1.1) was assayed following Marklund and Marklund (1974), and the ability of enzyme to auto oxidize epinephrine was recorded at 480 nm. Catalase activity (CAT; EC 1.11.1.6) was determined by Aebi (1984) and the disappearance of H_2_O_2_ was monitored at 240 nm for 3 min. The method of Bergmeyer (1974) was used for determination of the activity of POD (EC 1.11.1.7) and rate of guaiacol oxidation was monitored at 470 nm. Polyphenol oxidase (PPO/EC.1.10.3.1) activity was detected by a protocol of Lavid et al. (2001). The purpurogallin production was monitored at 495 nm and the enzyme activity was expressed in U mg^−1^ protein^−1^ min^−1^.

### 2.6 Statistical analysis

The results presented in the graphs are the means ± standard error of three replicates (n = 3). The results were statistically confirmed by analysis of variance (ANOVA). Tukey's HSD test was applied to find means are significantly different from each other at *p ≤ 0.05* level using Minitab 17 Statistical Software. Means that do not share a letter are significantly different at *p ≤ 0.05* significance level.

## 3 Results

### 3.1 Growth biomarkers

**Fig. 1:**
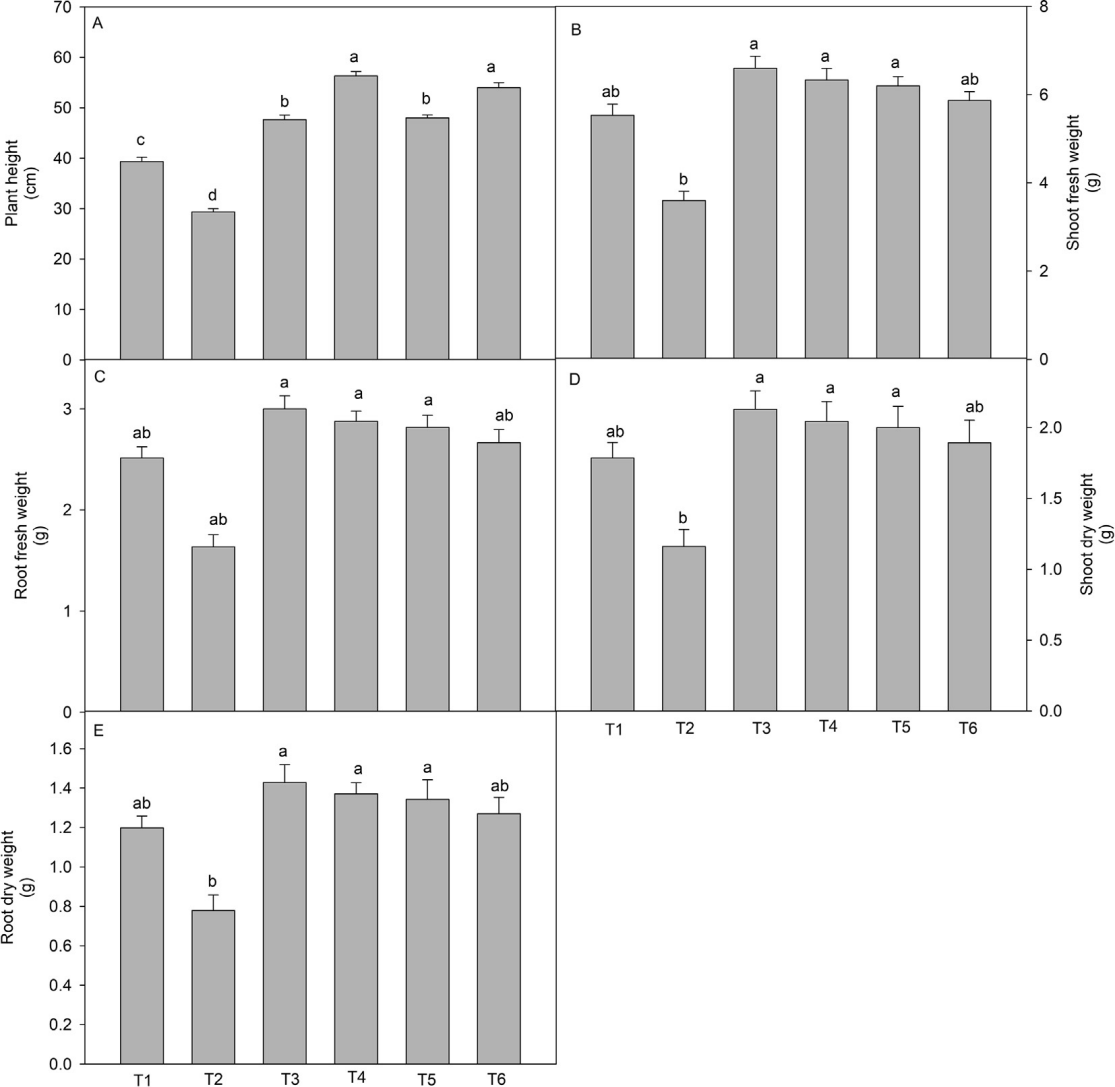
Effect of wuxal amino (2 and 3 cm L^−1^) under different mode (foliar and soil applied) on the salinity induced changes in (A) plant height, (B) shoot fresh weight, (C) Root fresh weight, (D) shoot dry weight, and (E) root dry weight of tomato plants at 60 days after planting. Data are means ± standard error of the three replicates (n = 3). Means that do not share a letter are significantly different at P ≤ 0.05 level according to Tukey’s test. [T1- Control; T2- NaCl (150 mM, through soil); T3- Wuxal amino (2 cm L^−1^_,_ through soil) + NaCl (150 mM, through soil); T4- Wuxal amino (2 cm L^−1^_,_ foliar spray) + NaCl (150 mM, through soil); T5- Wuxal amino (3 cm L^−1^_,_ through soil) + NaCl (150 mM, through soil); T6- Wuxal amino (3 cm L^−1^_,_ foliar spray) + NaCl (150 mM, through soil).

It is evident from the Fig. 1. that various growth parameters (plant height, fresh mass of shoot and root, and dry mass of shoot and root) were significantly affected by the treatments (Fig. 1A-E). The plants raised in the soil treated with 150 mM of NaCl showed significant loss of plant height and reduced the fresh and dry mass of shoot and root. Moreover, the loss of plant height (25.4%), shoot and root fresh mass (34.9% and 24.1%), and shoot and root dry mass (34.8% and 35.2%) in comparison to control plants. On the other hand, stressed plants treated with wuxal amino (2 cm L^-1^ and 3 cm L^-1^) through different modes i.e. soil and foliar spray showed promising recovery. On comparing two modes and concentrations, it was found that wuxal amino (2 cm L^-1^) through foliar spray successfully recovered the loss of plant height whereas; wuxal amino (2 cm L^-1^) through soil recovered the fresh and dry mass of shoot and root.

### 3.2 Physiological traits

**Fig. 2:**
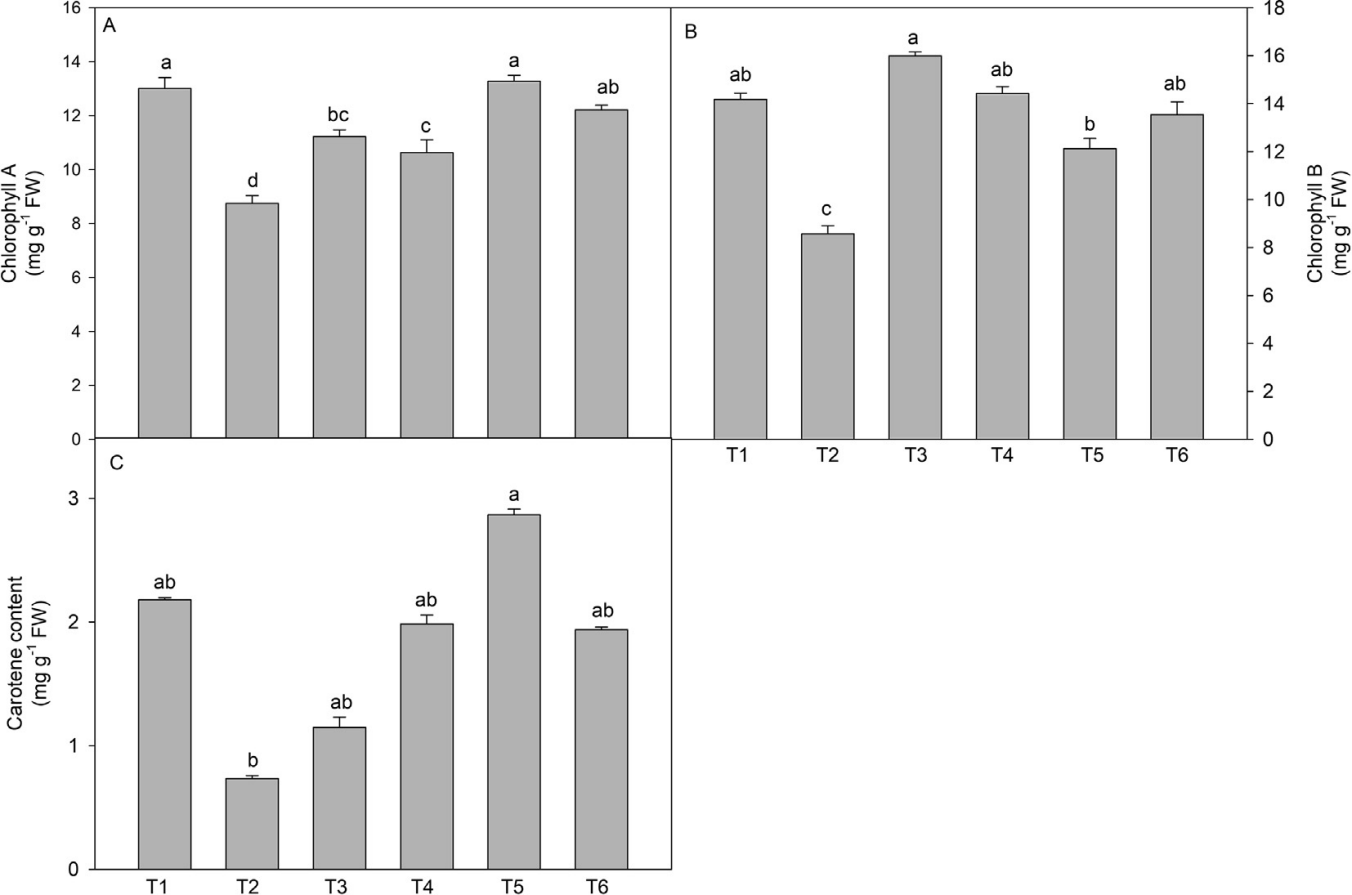
Effect of wuxal amino (2 and 3 cm L^−1^) under different mode (foliar and soil applied) on the salinity induced changes in (A) chlorphyll A, (B) chlorophyll *B*, and (C) carotene content of tomato plants at 60 days after planting. Data are means ± standard error of the three replicates (n = 3). Means that do not share a letter are significantly different at P ≤ 0.05 level according to Tukey’s test. [T1- Control; T2- NaCl (150 mM, through soil); T3- Wuxal amino (2 cm L^−1^_,_ through soil) + NaCl (150 mM, through soil); T4- Wuxal amino (2 cm L^−1^_,_ foliar spray) + NaCl (150 mM, through soil); T5- Wuxal amino (3 cm L^−1^_,_ through soil) + NaCl (150 mM, through soil); T6- Wuxal amino (3 cm L^−1^_,_ foliar spray) + NaCl (150 mM, through soil).

Physiological traits (Chl a, Chl b, and carotenoids) exhibited a decline in plants grown on the soil amended with 150 mM of NaCl. Out of three physiological traits, carotene contents showed maximum loss (66.5%) in comparison to control plants. However, when plants were treated with wuxal amino (2 and 3 cm L^-1^) through different modes, a promising recovery response in comparison to stressed plants was observed. Wuxal amino (3 cm L^-1^) through foliar spray successfully recovered the loss of Chl. a and carotene contents whereas wuxal amino (2 cm L^-1^) through soil recovered the loss of Chl. b (Fig. 2).

### 3.3 Stress biomarkers

**Fig. 3:**
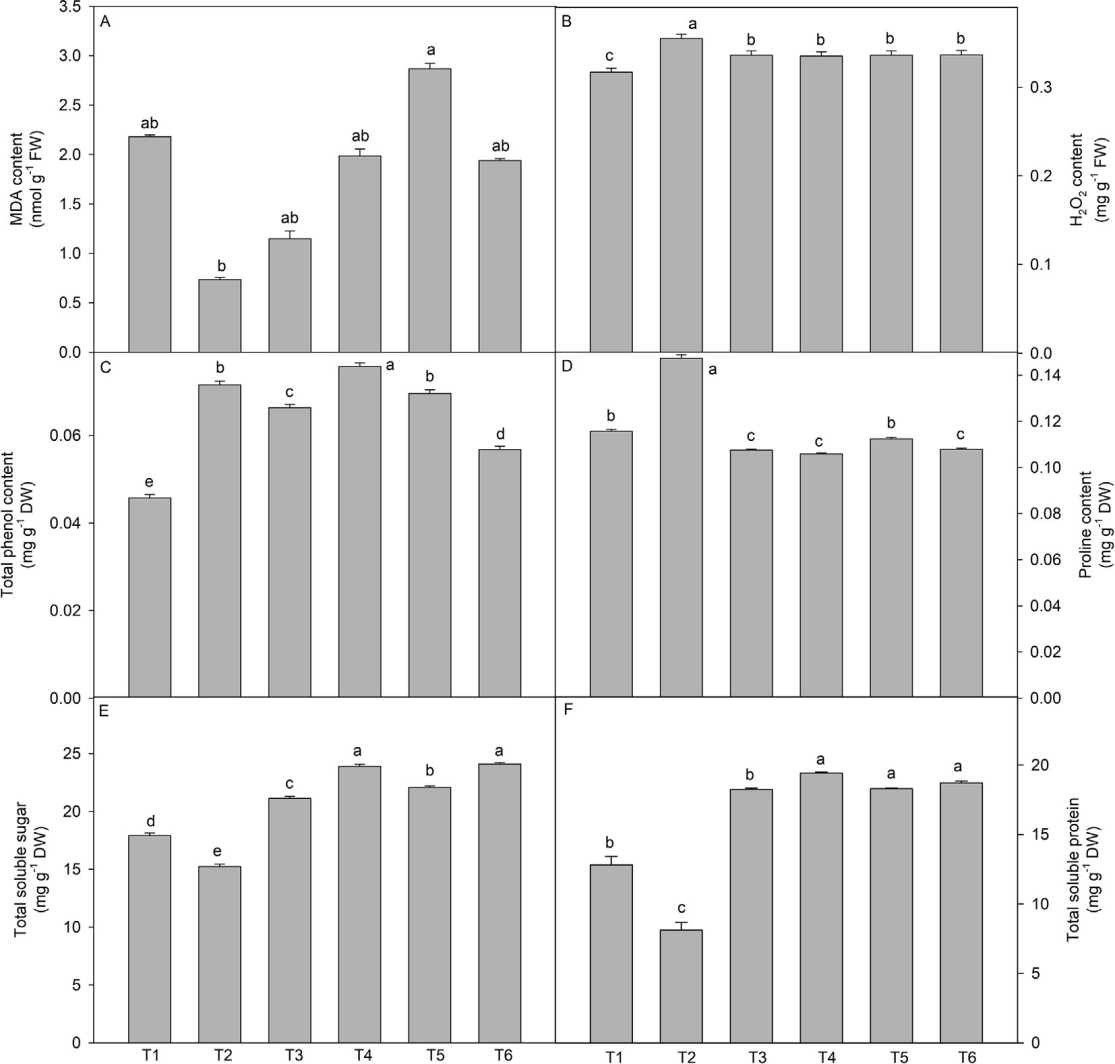
Effect of wuxal amino (2 and 3 cm L^−1^) under different mode (foliar and soil applied) on the salinity induced changes in (A) MDA content (B) H_2_O_2_ content (C) Total phenol content, (D) proline content, (E) total soluble sugar content, and (F) total soluble protein of tomato plants at 60 days after planting. Data are means ± standard error of the three replicates (n = 3). Means that do not share a letter are significantly different at P ≤ 0.05 level according to Tukey’s test. [T1- Control; T2- NaCl (150 mM, through soil); T3- Wuxal amino (2 cm L^−1^_,_ through soil) + NaCl (150 mM, through soil); T4- Wuxal amino (2 cm L^−1^_,_ foliar spray) + NaCl (150 mM, through soil); T5- Wuxal amino (3 cm L^−1^_,_ through soil) + NaCl (150 mM, through soil); T6- Wuxal amino (3 cm L^−1^_,_ foliar spray) + NaCl (150 mM, through soil).

The plants exposed to 150 mM of NaCl showed contrasting response for MDA and H_2_O_2_ content. It decreased MDA content whereas, enhanced the H_2_O_2_ content in comparison to non-treated control plants. However, wuxal amino (2 and 3 cm L^-1^) through different mode of application did not show any significant response for the H_2_O_2_ content whereas, 3 cm L^-1^ through soil significantly increased the MDA content but 2 cm L^–1^ through soil reduced the MDA content (Fig. 3A and B).

### 3.4 Total phenol content

The presence of NaCl (150 mM) in the soil caused a significant increase in the total phenol content in the plants compared to control plants (Fig. 3C). The plants grown under normal conditions showed minimum values for total phenol content in comparison to NaCl treated ones. However, total phenol content increased in wuxal amino treated plants and maximum values for total phenol content was observed in pants treated with wuxal amino (2 cm L^-1^) through foliar application then followed by wuxal amino (3 cm L^-1^) through soil (Fig. 3C).

### 3.5 Soluble sugar, soluble protein and proline contents

The soluble sugar and protein content increased significantly by the wuxal amino treatments irrespective of the concentration and mode of application over the control plants (Fig. 3E and F). However, the plants raised in the presence of NaCl (150 mM) showed reduction of total soluble sugar and proteins content, compared with the control. Furthermore, the plants raised from seeds treated with wuxal amino (2 cm L^-1^) through foliar application neutralised the damaging effect cause by NaCl (Fig. 3E and F).

The plants developed in the soil administered with NaCl, showed maximum accumulation of proline irrespective of the treatments (Fig. 3D). However, treatment of wuxal amino (2 and 3 cm L^-1^) through different mode of application showed similar response and it decreased the accumulation of proline in comparison to control plants.

### 3.6 Ascorbic acid

**Fig. 4:**
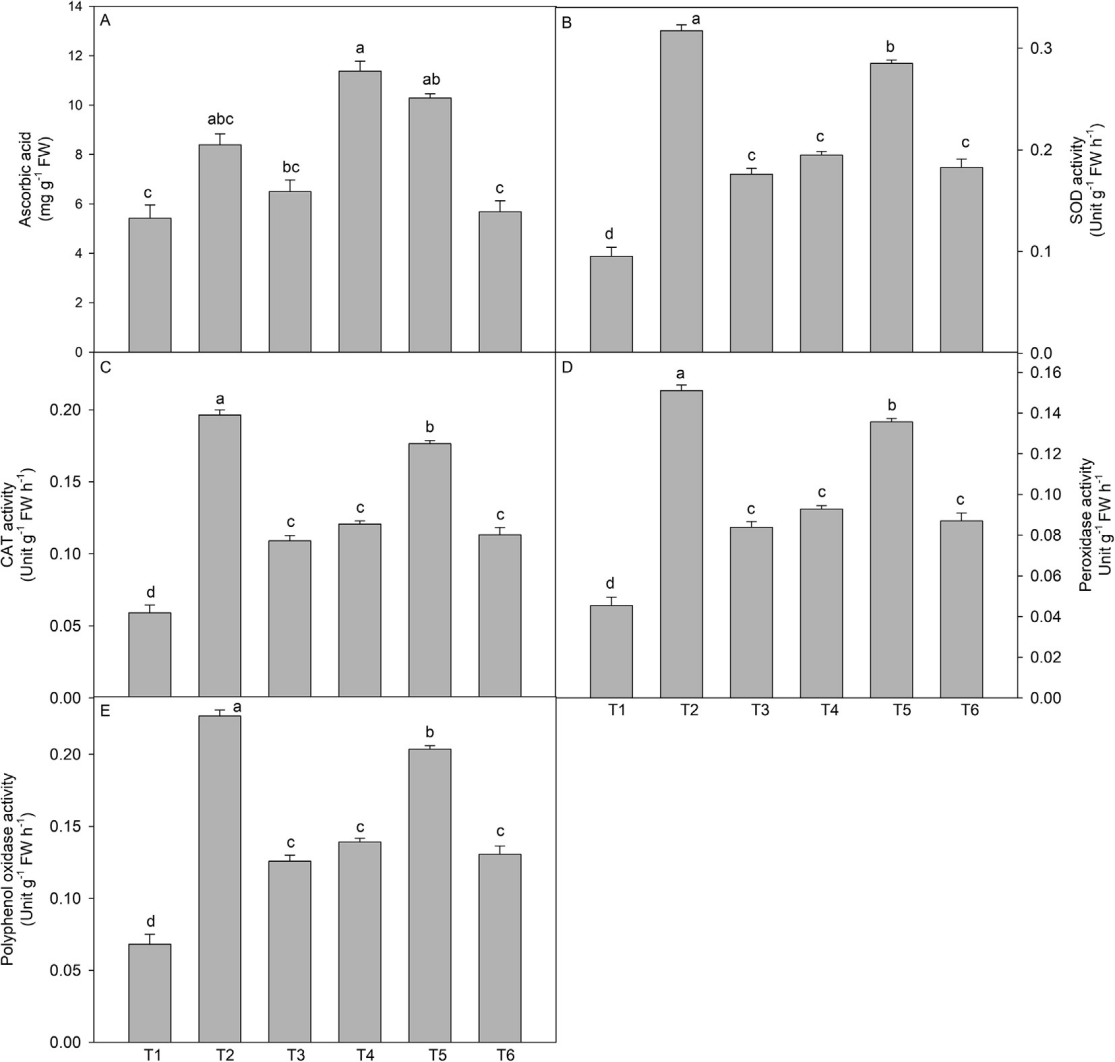
Effect of wuxal amino (2 and 3 cm L^−1^) under different mode (foliar and soil applied) on the salinity induced changes in (A) ascorbic acid (B) SOD activity (C) CAT activity, (D) peroxidase activity, and (E) polyphenol oxidase activity of tomato plants at 60 days after planting. Data are means ± standard error of the three replicates (n = 3). Means that do not share a letter are significantly different at P ≤ 0.05 level according to Tukey’s test. [T1- Control; T2- NaCl (150 mM, through soil); T3- Wuxal amino (2 cm L^−1^_,_ through soil) + NaCl (150 mM, through soil); T4- Wuxal amino (2 cm L^−1^_,_ foliar spray) + NaCl (150 mM, through soil); T5- Wuxal amino (3 cm L^−1^_,_ through soil) + NaCl (150 mM, through soil); T6- Wuxal amino (3 cm L^−1^, foliar spray) + NaCl (150 mM, through soil).

The leaves of plants treated with wuxal amino had higher ascorbic acid and the maximum content has been reported in the plants treated with wuxal amino (2 cm L^-1^) through foliage in comparison to control plants. Additionally, plants grown on the soil amended with NaCl (150 mM) also exhibited increased ascorbic acid but this increase was less than the wuxal amino (2 cm L^-1^; through foliage) and (3 cm L^-1^; through soil) treatments (Fig. 4A).

### 3.7 Antioxidant enzymes activities

#### 3.7.1 Activity of superoxide dismutase (SOD)

It is evident from graph 4B, that activity of SOD showed differential changes under various treatments. The maximum activity of SOD was reported in the plants grown under the influence of NaCl (150 mM) over the treatments. However, this suite followed by the application of wuxal amino (3 cm L^-1^) through soil and also proved best among other concentrations and mode of application.

#### 3.7.2 Activity of catalase

Plants grown on NaCl treated soil possessed higher CAT activity over the control plants. The activity of CAT showed dual response in the presence of wuxal amino and maximum increase was shown by the wuxal amino at concentration of 3 cm L^-1^ through soil and minimum due to 2 cm L^-1^ through soil (Fig. 4C).

#### 3.7.3 Peroxidase and polyphenol oxidase activity

It is evident from Fig. 4D and E that a significant increase in the activity of peroxidase and polyphenol oxidase in response to the NaCl and/or wuxal amino was observed. Control plants possessed minimum activity of peroxidase and polyphenol oxidase. Moreover, the application of wuxal amino at 3 cm L^-1^ to NaCl treated plants significantly increased the activity of peroxidase and polyphenol oxidase in comparison to control plants (Fig. 4D and E).

## 4 Discussion

Salinity stress is considered as one of the most damaging abiotic factors for crop growth and loss of yield. Efficiency of several management techniques has been exploited to lessen the damaging effects by mediating either quick removal of toxic ions from the soil solution or their sequestration into the less sensitive organelles concomitant with strengthening of the existing tolerance mechanisms. In this connection, the introduction of novel mitigating agents can improve the growth and yield of plant species under saline conditions. Despite the extensive study of NaCl, two essential unaddressed questions remain: salt-alkaline stress (SAS) and salt-alkali stress (SAS). Salinization and alkalization can often occur together and in the process causing extensive harm (Kawanabe and Zhu, 1991). Salt stress mainly causes water deficiency and ion toxicities (Munns, 2002, Parida and Das, 2005, Munns et al., 2020). Alkali-stress is induced by the same stress factors as salt stress, but the influence of high pH stress is added. The high pH environment surrounding the roots can directly cause other ions (e.g. Ca^2+^, Mg^2+^, and others) to precipitate (Shi and Sheng, 2005). High pH can also result in a loss of protons, the degradation or suppression of transmembrane electrochemical potential gradients in plant roots, as well as the loss of normal physiological root functions including ion uptake and water absorption (Yang et al., 2007). Under salinity stress, plant survival depends not only on the ability to cope with water stress and ion toxicity, but also on high pH tolerance.

In the present study a novel supplement, wuxal amino was tested for its effectiveness on mitigating the damaging effects of salinity on tomato plants. Wuxal amino is an organic bio-stimulant contains 9% nitrogen not only rich in essential amino acids like proline, alanine, threonine, and glycine but also buffering agents used to improve the quality of liquid fertilizers for increase its effectiveness when it combines with pesticides and micronutrients (Liu et al., 2016). It is vital for plants to adjust the extracellular pH that damages the roots ability to resist salt stress (Yang et al., 2009). In the present study, both foliar and root applied wuxal amino showed ameliorative effect in growth inhibition at lower concentrations (2 cm/L). Growth in terms of plant height, root length, fresh and dry weight was significantly enhanced due to application of wuxal amino under salinity stress. Salinity drastically declined the plant height and weight in present study that corroborates with the results of (Chung et al., 2019, Hasan et al., 2020, Soliman et al., 2020b). The decline in morphological attributes and biomass accumulation due to salinity stress is cumulative effect on the key metabolic and assimilatory attributes like uptake, transport and assimilation of mineral elements (Ahanger and Agarwal, 2017, Ahanger et al., 2019, Osman et al., 2020), enzyme functioning (Elkelish et al., 2020), photosynthesis and redox homeostasis (Ahanger et al., 2019, Shah et al., 2020). All these interruptions are regulated genetically (Ogawa et al., 2011) besides stresses hampering the cell cycle progression (Qi and Zhang, 2020). Using Bio stimulants for improving growth has been proposed as promising management technique for crop improvement (Yakhin et al., 2017).

Reports on the beneficial effects of Wuxal amino on plants are very rare. In the proposed technique, the application of wuxal amino significantly increased the levels of chlorophyll as well as improved plant growth under salinity stress. Synthesis of pigments can increase the production of energy and serve as a source for necessary cellular functions (Ali and Ashraf, 2011, Li et al., 2018). Decline in photosynthesis and growth under salinity results directly from the excess accumulation of Na altering the integrity of photosystems hence lessening their performance (Osman et al., 2020, Yang et al., 2020). Recently Kwon et al. (2019) have demonstrated declined chlorophyll synthesis in salt stressed *Dianthus caryophyllus* resulting in reduction in photosynthesitic and hence growth. However, in present study it was observed that application of wuxal amino proved beneficial in enhancing the synthesis of chlorophyll pigments. The observed improvement in the photosynthetic pigments by WUXAL Amino is an organic bio-stimulant and contains 9% organically fixed nitrogen, which is totally offered to plants. WUXAL Amino contains amino acids (648 g/l) as well as polypeptides. Increased synthesis of carotenoids due to wuxal application may have contributed to photosynthetic protection by mediating ROS scavenging and contributing to redox maintenance (Hashimoto et al., 2016). Increased synthesis of chlorophyll and carotenoids due to application of biostimulators have been reported by Alam et al., 2014, Szabó et al., 2014 in *Tagetes Erecta* and *Prunus mahaleb* respectively. However, the effectivity of wuxal amino biostimulator under salinity stress is largely unknown.

It was observed that the activity of antioxidant enzymes like SOD, CAT, POD and PPO significantly higher due to salinity stress. Such enhancement in the activities of antioxidants have been reported by others as well (AbdElgawad et al., 2016, Elkelish et al., 2019, Alhaithloul et al., 2020, Soliman et al., 2020c, Zaheer et al., 2020). Up-reguated antioxidant functioning assists to counteract the damaging effects of salinity induced oxidative damage to membranes, lipids and proteins (Ellouzi et al., 2011, Ahanger and Agarwal, 2017). Application of wuxal amino bio-stimulant resulted in further enhancement in the activities of the antioxidant enzymes. SOD provides first line defence against the toxic superoxide radical thereby leading to protection of major cellular pathways like photosynthetic electron transport (Abdallah et al., 2016, AbdElgawad et al., 2016). In addition, the optimal functioning of other enzymes like CAT or any other H_2_O_2_ scavenging enzyme is important for oxidative damage amelioration (Zhou et al., 2018). It was interesting to observe that activity of CAT and POD increased significantly due to application wuxal amino. Earlier the application of bio-stimulant has been reported to improve the antioxidant functioning of plants like tomato (Boeckx et al., 2015, Sidhu et al., 2017) and bean (Kocira et al., 2020). Reports regarding the influence of wuxal amino bio-stimulant on the antioxidant functioning are not available therefore present study makes an important mark towards the sustainable approach for crop stress tolerance. ROS including H_2_O_2_, O_2_^–^, OH etc are extremely dangerous for normal cellular functioning and their quick elimination due to up-regulation of antioxidant system significantly contributes to plant growth regulation under extreme conditions (Zhou et al., 2018, Ahmad et al., 2019b, Hasanuzzaman et al., 2019). Up-regulated functioning of antioxidant enzymes contributes to protection of photosynthesis, enzyme functioning and membrane protection hence plant performance is maintained (Huseynova, 2012, Yakhin et al., 2017, Ahanger et al., 2019). Both modes of application showed significant effect however foliar applied wuxal proved more beneficial at both concentrations. Peroxidases mediate elimination of H_2_O_2_ at membranes while as CAT neutralises cytosolic H_2_O_2_ making cellular functioning to continue uniformly (Ahmad et al., 2010, Das and Roychoudhury, 2014, Ahmad et al., 2019, Kohli et al., 2019). In addition to this the content of ascorbic acid was significantly improved due to wuxal application thereby protecting the cellular functioning and hence contributing to plant performance. Ascorbic acid and glutathione form a typical component of key ROS scavenging pathway– the ascorbate glutathione pathway operating in chloroplast and mitochondria for better energy generation and hence plant functioning (Akram et al., 2017). Ascorbic acid is essential non-enzymatic antioxidant helping in redox homeostasis maintenance, enzyme functioning and stress tolerance (Dolatabadian and Jouneghani, 2009, Das and Roychoudhury, 2014, Shereefa and Kumaraswamy, 2016, Osman et al., 2020). Besides this, the application of wuxal amino resulted in increased accumulation of phenol content which was correlated with increased PPO activity in the present study. Increased PPO activity mediates oxidation of phenolic compounds to reactive o-quinones that interact with oxygen and proteins (Boeckx et al., 2015). PPO has both pro- as well as antioxidant functioning. Phenolic compounds have key roles in cell division, hormonal and photosynthetic regulation, nutrient mineralization and reproduction, and can have potential role in stress signalling (Sharma et al., 2019). Application of weed extract rich in amino acid significantly improved the antioxidant functioning in bean thereby contributing to enhanced accumulation of phenols, flavonoids and anthocyanins (Kocira et al., 2020).

Secondary metabolites like phenols can also contribute to osmoregulation and strengthen the effect of compatible osmolytes like proline. Greater accumulation of compatible solutes is an important strategy to counteract the damaging effects of stresses including salinity. Salinity mediated accumulation of proline observed in present study corroborate with earlier findings (Hmidi et al., 2018, Soliman et al., 2020a). Proline accumulation assists in osmoregulation, stabilization of key cellular structures and their functioning, enzyme functioning, ROS scavenging and redox homeostasis maintenance (Meena et al., 2019, Osman et al., 2020). Wuxal mediated enhancement in the proline content may have contributed to growth and cellular functioning maintenance through greater stabilization of cellular structures and enzyme functioning involved in key metabolic pathways (Yang et al., 2009). The accumulated proline may distribute in the cytoplasm to balance the osmotic pressure from vacuoles and to protect biomacromolecules. Under salinity stress, proline accumulation depends on the alkali-resistant traits of plant (Shi and Sheng, 2005, Yang et al., 2007). Buffering agent is closely correlated with the mechanism of plant resistance to salt stress. Increased proline accumulation has been reported to protect photosynthesis by actively protecting the functioning of Rubisco (Soshinkova et al., 2013, Azooz et al., 2015, Abdel Razik et al., 2020). Accumulation of proline is regulated by modulation of the gene functions involved in its synthesis and catabolism (Kovács et al., 2019). Reports discussing the effect of wuxal amino on proline accumulation under salinity are not available therefore further studies are required to study the exact mechanisms involved. Besides this, wuxal application induced the synthesis of proteins thereby lessening the salinity mediated decline in protein accumulation. Plants improve the expression of genes coding for specific proteins involved in regulation of various functions (Witzel et al., 2009, Razzaque et al., 2019, Qin et al., 2021).

## 5 Conclusion

It is concluded that salt administered through soil triggered oxidative damage and resulted into the reduced growth and declined physiological performance. Application of wuxal amino to salt stressed tomato plants either through foliage or through soil induced ameliorative response owing to pH adjustment and up-regulation of antioxidant enzymes. Beside this, plants treated with wuxal amino also showed enhanced accumulation of osmoprotectant (proline) and phenols that also act as a scavenging tool to remove the excess ROS under salt stress. This study advocates the beneficial use of wuxal amino application in protecting tomato plants under salinity stress, however, further studies are required to unravel actual mechanisms.

## Declarations

**Ethics approval**: Not Applicable.

**Consent to participate**: All authors consent to participate in this manuscript.

**Consent for publication**: All authors consent to publish this manuscript in Saudi Journal of Biological Science.

**Availability of data and material**: Data will be available on request to corresponding or first author.

**Code availability**: Not Applicable.

## Author contributions

MMA, MSO and MSA drafted the experimental design and SME performed the experiments. MY, MH, KJ, KH and MSO helped in data collection, data analysis and initial draft of manuscript text. All authors read the manuscript before communication.

## Declaration of Competing Interest

The authors declare that they have no known competing financial interests or personal relationships that could have appeared to influence the work reported in this paper.
